# A Gut-Wrenching Feeling: Overcoming Cognitive Biases in an Atypical Presentation of Chronic Mesenteric Ischemia

**DOI:** 10.7759/cureus.36416

**Published:** 2023-03-20

**Authors:** Mohammad A Ahmed-Khan, Kayvon Moin, Mahnoor Hanif, Mohamed Jiffry, Jonathan Vargas, Tooba Z Khan, Samina Khan, Arezou Nazary

**Affiliations:** 1 Department of Internal Medicine, University of Vermont, Burlington, USA; 2 Department of Internal Medicine, Danbury Hospital - Yale School of Medicine, Danbury, USA; 3 School of Medicine, American University of the Caribbean, Cupecoy, SXM; 4 Medicine and Surgery, CMH Lahore Medical College and Institute of Dentistry, Lahore, PAK; 5 Dentistry, CMH Lahore Medical College and Institute of Dentistry, Lahore, PAK; 6 Department of Internal Medicine, University of Alberta, Edmonton, CAN; 7 Department of Internal Medicine, Connecticut Institute for the Communities, Danbury, USA

**Keywords:** s:malnutrition, non-bloody diarrhea, git endoscopy, yersinia enterocolitica, chronic mesenteric ischemia

## Abstract

Chronic mesenteric ischemia is a rare but serious condition that can present with a variety of symptoms, including abdominal pain, diarrhea, and weight loss. Our case report presents a 63-year-old male with a past medical history of generalized anxiety disorder, Barrett's esophagus, hypertension, hyperlipidemia, chronic obstructive pulmonary disease (COPD) with active smoking who initially presented with severe diffuse abdominal pain, nausea, vomiting, and chronic diarrhea resulting in malnutrition and 40-pound weight loss over a six-month span.

The patient underwent extensive diagnostic evaluation and was diagnosed with Yersinia gastroenteritis via gastroenteritis panel (GI Panel), explaining all of the patient's symptoms. The patient underwent treatment for said gastroenteritis but did not experience remission of symptoms, leading to further diagnostic evaluations; a definitive diagnosis was not found, yet the patient's symptoms persisted. The patient then underwent extensive serologic and endoscopic evaluation, after extensive imaging and diagnostic work-up, the patient was finally diagnosed with chronic mesenteric ischemia (CMI) of the superior mesenteric artery (SMA) with severe celiac and inferior mesenteric artery (IMA) stenosis. The patient initially underwent stenting (7 mm by 26 mm Balloon Mounted Lifestream^TM^ Covered Stent; Becton Dickson (BD); Franklin Lakes, NJ, USA), which provided temporary relief to his symptoms, however, the relief did not last long. Upon reimaging, the patient was found to have stenosis of the stent, leading to the eventual placement of a bare-metal stent (Express^TM^ LD 7 x 27 mm balloon mounted bare-metal stent; Boston Scientific; Boston, MA, USA) across the celiac artery as well as the placement of an IMA stent (Innova^TM^ Self-expanding 5 x 20 mm bare-metal stent; Boston Scientific). This eventually resulted in the resolution of the patient's symptoms, eventual weight gain, and improvement in quality of life.

## Introduction

Chronic mesenteric ischemia (CMI) is a rare but potentially life-threatening condition characterized by inadequate blood supply to the intestines, leading to chronic abdominal pain, diarrhea, and weight loss [1]. It is typically caused by atherosclerotic stenosis or occlusion of one or more mesenteric arteries, most commonly the superior mesenteric artery [1]. Although CMI is a well-established clinical entity, its diagnosis can be challenging due to its diverse clinical presentation, which often mimics other gastrointestinal disorders.

## Case presentation

The patient in this case is a 63-year-old male with a past medical history of generalized anxiety disorder, Barrett's esophagus, hypertension, hyperlipidemia, and chronic obstructive pulmonary disease (COPD) with active smoking. He presented to the hospital complaining of severe diffuse abdominal pain, nausea, vomiting, and chronic diarrhea. He reported experiencing six to seven episodes of non-bloody, watery diarrhea daily, leading to malnutrition and a 40-pound weight loss over a six-month span.

The patient had previously presented with the same chief complaint some months prior when he was diagnosed with Yersinia gastroenteritis. A Computed Tomography (CT) of his abdomen and pelvis revealed mild jejunal wall thickening consistent with enteritis and he was treated with levofloxacin. Despite the antibiotic regimen, the patient's symptoms of severe abdominal pain, nausea, and chronic diarrhea persisted. A Magnetic Resonance Imaging (MRI) of the abdomen and pelvis was eventually obtained, but it did not aid in a specific diagnosis. He was then discharged and followed closely by gastroenterology in the ensuing months.

During his follow-up visits, endoscopy revealed esophageal mucosal changes consistent with short-segment Barrett's esophagus and a small hiatal hernia without gross lesions in the stomach and duodenum. Colonoscopy revealed erythematous mucosa in the terminal ileum and a solitary ulcer in the cecum. Biopsies from the right and left colon with subsequent pathology reports were negative for inflammatory disorders.

**Figure 1:**
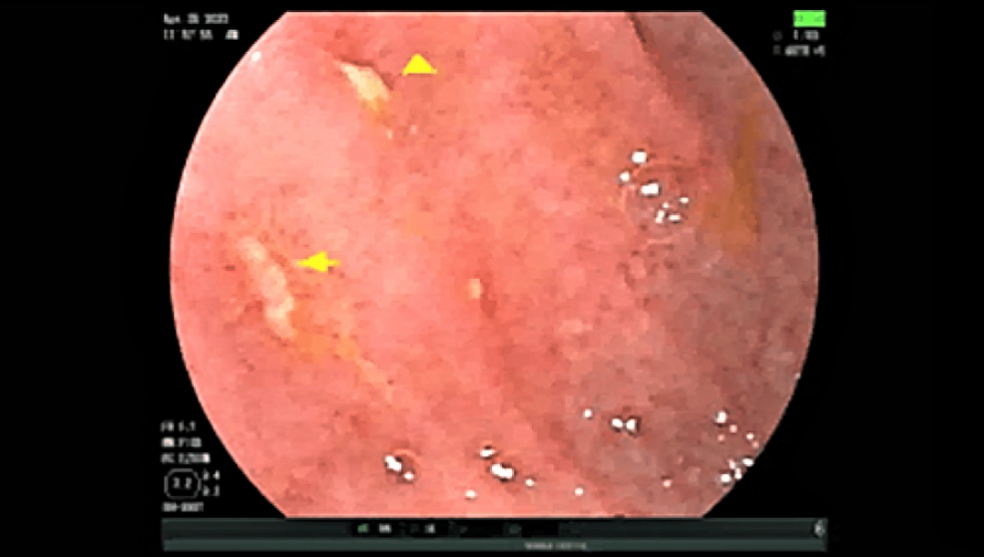
Images of the jejunum on balloon enteroscopy showing presence of multiple non-bleeding ulcers (marked by yellow arrows)

To further investigate the cause of his symptoms, the patient underwent a capsule endoscopy and eventually a double-balloon enteroscopy. The capsule endoscopy was remarkable for mild gastritis with patchy areas of erythema, erosions, and ulcers in the jejunum and distal ileum. The double-balloon enteroscopy revealed many non-bleeding superficial jejunal ulcers in the proximal and mid jejunum, the largest lesion being 5 mm with mild mucosal changes (Figure 1). Biopsies were taken, and pathology reports again showed no remarkable findings.

During his readmission, the patient was malnourished with significant weight loss. Extensive laboratory testing was performed to rule out infectious, ischemic, inflammatory, autoimmune, and vascular etiologies, including antinuclear antibody (ANA) titer, myeloperoxidase antibodies, proteinase antibodies, cryoglobulin, syphilis, hepatitis, GI panel, tick-borne organisms including Lyme, Babesia, and Anaplasma, and human immunodeficiency virus (HIV). The patient's labs were significant for elevated calprotectin, positive fecal lactoferrin, and elevated C-reactive protein (CRP).

**Figure 2:**
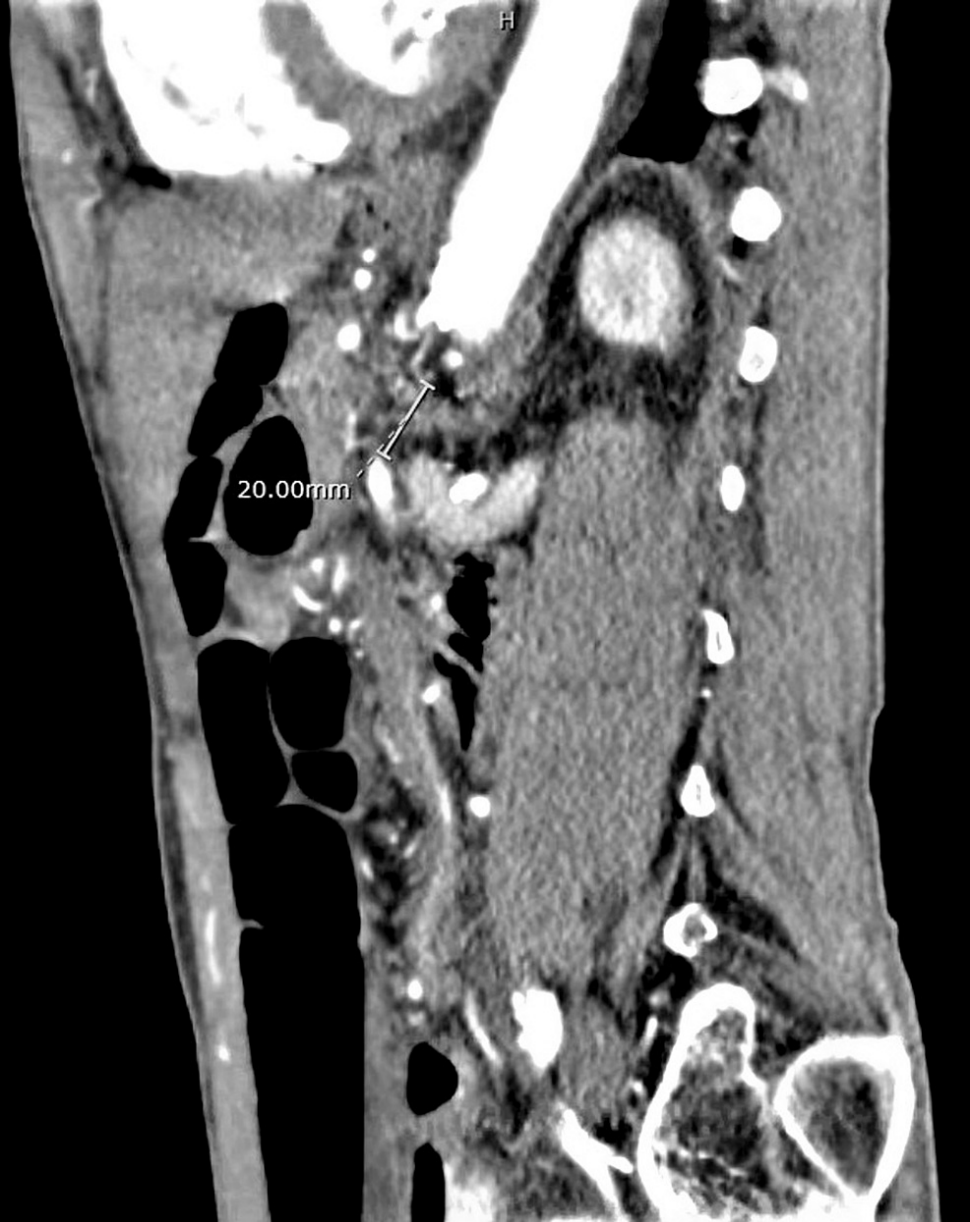
CT Angiography showing a 20 mm occlusion at the origin of the superior mesenteric artery

**Figure 3:**
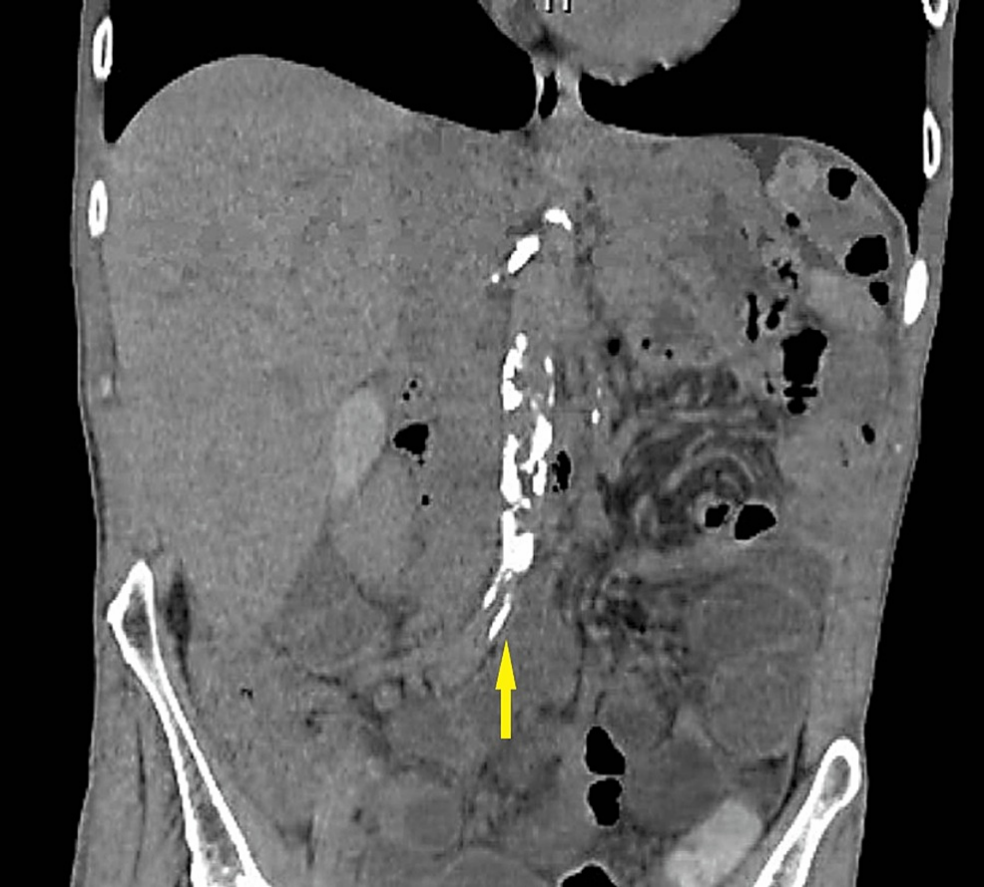
Severe atherosclerotic plaque burden of the aorta found on CT angiography (demarked by following the yellow arrow superiorly)

A repeat CT scan of the abdomen and pelvis with contrast was obtained, which revealed mild diffuse abnormal wall thickening involving the terminal ileum and cecum. The CT also revealed CMI of the superior mesenteric artery (SMA) due to a 2 cm origin (Figure 2) occlusion with distal reconstitution and severe atherosclerotic burden of the aorta (Figure 3). Findings were also significant for severe celiac and inferior mesenteric artery (IMA) origin stenosis. The mild diffuse abnormal wall thickening involving the terminal ileum and cecum remained unchanged, raising concerns for ischemic colitis.

**Figure 4:**
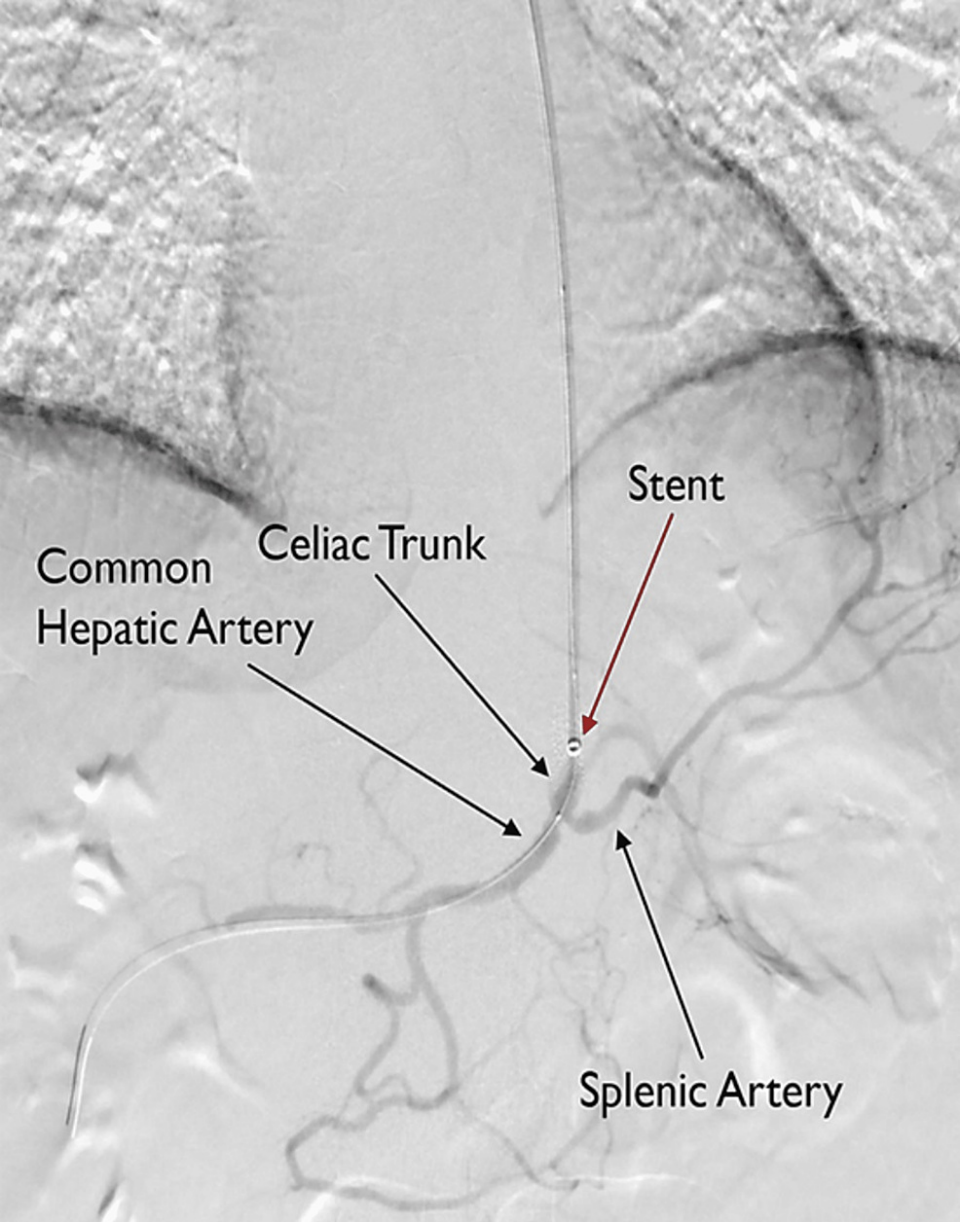
Imaging of the placement of balloon-mounted bare metal stent within the patient’s previous celiac covered stent as well as a self-expanding bare metal stent in the inferior mesenteric artery.

Vascular surgery was consulted, and the patient received a celiac stent (7 mm by 26 mm Balloon Mounted Lifestream^TM^ Covered Stent; Becton Dickson (BD); Franklin Lakes, NJ, USA) (Figure 4). Within 48 hours, the patient's symptoms were relieved, and he was discharged. However, at his one-month follow-up, the patient's symptoms returned, and a CT angiogram was repeated, revealing a significant stenosis at the mid portion/distal celiac artery stent. Therefore, the patient underwent an aortogram, balloon angioplasty of the celiac and IMA, placement of a bare-metal stent across the celiac artery stent (Express^TM^ LD 7 x 27 mm balloon mounted bare-metal stent; Boston Scientific; Boston, MA, USA), as well as placement of an IMA stent (Innova^TM^ self-expanding 5 x 20 mm bare-metal stent; Boston Scientific). The patient has since been appropriately gaining weight and no longer has abdominal pain or diarrhea.

## Discussion

CMI is a condition secondary to insufficient blood flow to the splanchnic circulation of the gastrointestinal tract [1]. This is usually in the setting of atherosclerotic stenosis [1] and its incidence has been noted in single and multiple vessel involvement of 17.5 to 18% of elderly patients [2]. Atherosclerosis is the most common cause of CMI; however, aortic dissection, vasculitis, fibromuscular dysplasia, radiation, and even cocaine abuse have been described as etiologies. Predisposing factors can include metabolic diseases such as diabetes mellitus, hyperlipidemia, hypercholesterolemia, and hypertension [2,3]; many of these aforementioned predisposing factors were present in our patient. It is interesting to note that CMI was actually first described in 1918 by Goodman [4] as a kind of abdominal angina due to the fitting description of the relapsing and remitting style of pain in relationship with meals that occurs in these patients [1,4].

Our patient's atypical presentation combined with a superimposed infection proposed a diagnostic dilemma in finding the appropriate final diagnosis. Our patient's symptomatology included nonspecific abdominal pain, chronic diarrhea, and weight loss [5]; evidently, a retrospective study by Barret et al. [6] showed that 98% of CMI patients had postprandial abdominal pain, 53.1% had weight loss, and 24.5% had diarrhea [2]. It is easy to look at the data and assume that the diagnosis was missed based on some sort of oversight as these are three cardinal symptoms of our presenting condition. However, these three symptoms are quite nonspecific and very common in gastrointestinal diseases such as a Yersinia infection [7], which our patient happened to also have. This combined with the fact that the work-up was spearheaded by the gastroenterology team whose other differential was inflammatory bowel disease allowed the primary team to fall prey to an "availability heuristic" [8]. An availability heuristic can be defined as a decision-making framework on the basis of a cognitive bias that allows clinicians to make quick but sometimes incorrect assessments which may also be traced back to the way most medical students learn early on by way of "buzz-words" [9]. In the setting of our patient's presentation, the Yersinia infection as well as an elevated fecal calprotectin and CRP elevation all pointed our diagnoses in directions other than CMI, though, in some cases, CRP and fecal calprotectin had been elevated. [10] This gap in reasoning can be explained by the manner of the patient's presentation as well as the slew of associated symptoms that further “muddied” the waters, driving the diagnoses to be discovered later in the clinical course.

The nonspecific nature of our patient's presentation and unclear diagnostic work-up, combined with a recently cleared Yersinia infection posed a puzzling picture for our team. With the patient's unrevealing serologic evaluation, and GI evaluation questionable for ulcerative disease, a tentative diagnosis of inflammatory bowel disease was given. However, once the pathology results came back negative, we were left with no other known modalities of endoscopic evaluation of diarrheal diseases [6,11]. We then opted to repeat a CT scan with intravenous contrast revealing atherosclerotic disease burden [12,13] and this allowed us to discover that the patient was suffering from CMI.

The patient was referred to vascular surgery for stenting as the optimal treatment modality [6], based on the study done on single-stent vs two-vessel stent placement it did not show outcomes being different and that was the treatment modality that we had opted for in our patient [14]. However, when our patient presented with recurrent symptoms and repeat imaging concerning for restenosis (which is possible in a CMI patient with recurrence of symptoms [15]), we decided to perform balloon angioplasty and repeat endovascular intervention; this seemed to be consistent with the data presented by Kougias et al. [16] in their review of 328 patients that underwent endovascular intervention. Their findings showed that balloon angioplasty with stenting had better patient outcomes than stenting alone in terms of restenosis rates; unfortunately their data was not statistically significant so we may need further studies to better corroborate their findings. We can possibly attribute our patient’s diagnosis of restenosis to the lack of his lifestyle changes, such as his continuation to smoke after extensive counseling.

## Conclusions

This case report's objective was to highlight the importance of considering CMI as a differential diagnosis for persistent abdominal pain with chronic diarrhea. Despite its lower likelihood at an initial presentation, CMI should be on every clinician's differential diagnosis. We also wanted to highlight the diagnostic dilemma that this condition poses due to its symptomatological overlap with other abdominal pathologies such as inflammatory bowel disease and infectious gastroenteritis We hope to emphasize the need for thorough diagnostic evaluation to accurately diagnose and treat patients with this rare but serious condition. Early recognition and appropriate treatment of CMI are essential in preventing long-term morbidity and mortality in these patients with non-specific presentations. Our hope is for clinicians to avoid decision-making heuristics that can sway them from the appropriate diagnosis. 

## References

[1] Wilson DB, Mostafavi K, Craven TE, Ayerdi J, Edwards MS, Hansen KJ (2006). Clinical course of mesenteric artery stenosis in elderly americans. Arch Intern Med.

[2] Hohenwalter EJ (2009). Chronic mesenteric ischemia: diagnosis and treatment. Semin Intervent Radiol.

[3] Hansen KJ, Wilson DB, Craven TE (2004). Mesenteric artery disease in the elderly. J Vasc Surg.

[4] Mahajan K, Osueni A, Haseeb M (2022). Abdominal Angina. https://www.ncbi.nlm.nih.gov/books/NBK441943/.

[5] Sreenarasimhaiah J (2005). Chronic mesenteric ischemia. Best Pract Res Clin Gastroenterol.

[6] Barret M, Martineau C, Rahmi G (2015). Chronic mesenteric ischemia: a rare cause of chronic abdominal pain. Am J Med.

[7] Aziz M, Yelamanchili VS (2022). Yersinia enterocolitica. https://www.ncbi.nlm.nih.gov/books/NBK499837/.

[8] Yeoh SW (2016). A delayed diagnosis of chronic mesenteric ischaemia: the role of clinicians’ cognitive errors. Case Rep Gastroenterol.

[9] Redelmeier DA, Ng K (2020). Approach to making the availability heuristic less available. BMJ Qual Saf.

[10] Gravito-Soares M, Gravito-Soares E, Figueiredo P, Tomé L (2018). Acute-on-chronic mesenteric ischaemia by early and diffuse atherosclerosis in a young adult patient. BMJ Case Rep.

[11] Kinoshita Y, Ariyoshi R, Fujigaki S, Tanaka K, Morikawa T, Sanuki T (2022). Endoscopic diagnosis of chronic diarrhea. DEN Open.

[12] Bala M, Catena F, Kashuk J (2022). Acute mesenteric ischemia: updated guidelines of the World Society of Emergency Surgery. World J Emerg Surg.

[13] Cognet F, Ben Salem D, Dranssart M (2002). Chronic mesenteric ischemia: imaging and percutaneous treatment. Radiographics.

[14] Malgor RD, Oderich GS, McKusick MA (2023). Results of single- and two-vessel mesenteric artery stents for chronic mesenteric ischemia. Ann Vasc Surg.

[15] Sharafuddin MJ, Olson CH, Sun S (2003). Endovascular treatment of celiac and mesenteric arteries stenoses: applications and results. J Vasc Surg.

[16] Kougias P, El Sayed HF, Zhou W, Lin PH (2007). Management of chronic mesenteric ischemia. The role of endovascular therapy. J Endovasc Ther.

